# Immunization in women’s lives: present and future

**DOI:** 10.61622/rbgo/2024FPS10

**Published:** 2024-10-15

**Authors:** Agnaldo Lopes da Silva, Ana Karolina Barreto Berselli Marinho, André Luis Ferreira Santos, Angelina Farias Maia, Cecilia Maria Roteli-Martins, César Eduardo Fernandes, Fabiola Zoppas Fridman, Giuliane Jesus Lajos, Isabella Ballalai, Juarez Cunha, Julio Cesar Teixeira, Márcia Marly de Medeiros, Manoel Afonso Guimarães Gonçalves, Monica Levi, Nilma Antas Neves, Renata Robial, Renato de Ávila Kfouri, Susana Cristina Aidé Viviani Fialho, Valentino Magno

**Affiliations:** 1 Universidade Federal de Minas Gerais Belo Horizonte MG Brazil -Universidade Federal de Minas Gerais, Belo Horizonte, MG, Brazil.; 2 Hospital das Clínicas Faculdade de Medicina Universidade de São Paulo São Paulo SP Brazil -Hospital das Clínicas, Faculdade de Medicina da Universidade de São Paulo, São Paulo, SP, Brazil.; 3 Universidade de Taubaté Taubaté SP Brazil -Universidade de Taubaté, Taubaté, SP, Brazil.; 4 Universidade Federal de Pernambuco Recife PE Brazil -Universidade Federal de Pernambuco, Recife, PE, Brazil.; 5 Faculdade de Medicina do ABC Santo André SP Brazil -Faculdade de Medicina do ABC, Santo André, SP, Brazil.; 6 Faculdade de Medicina do ABC Santo André SP Brazil -Faculdade de Medicina do ABC, Santo André, SP, Brazil.; 7 Hospital Femina Porto Alegre RS Brazil -Hospital Femina, Porto Alegre, RS, Brazil.; 8 Universidade Estadual de Campinas Campinas SP Brazil -Universidade Estadual de Campinas, Campinas, SP, Brazil.; 9 Sociedade Brazileira de Imunizações São Paulo SP Brazil -Sociedade Brazileira de Imunizações, São Paulo, SP, Brazil.; 10 Sociedade Brazileira de Imunizações São Paulo SP Brazil -Sociedade Brazileira de Imunizações, São Paulo, SP, Brazil.; 11 Universidade Estadual de Campinas Campinas SP Brazil -Universidade Estadual de Campinas, Campinas, SP, Brazil.; 12 Instituto Tropical de Medicina Reprodutiva Cuiabá MT Brazil -Instituto Tropical de Medicina Reprodutiva, Cuiabá, MT, Brazil.; 13 Pontifícia Universidade Católica do Rio Grande do Sul Porto Alegre RS Brazil -Pontifícia Universidade Católica do Rio Grande do Sul, Porto Alegre, RS, Brazil.; 14 Sociedade Brazileira de Imunizações São Paulo SP Brazil -Sociedade Brazileira de Imunizações, São Paulo, SP, Brazil.; 15 Universidade Federal da Bahia Salvador BA Brazil -Universidade Federal da Bahia, Salvador, BA, Brazil.; 16 Hospital das Clínicas Faculdade de Medicina Universidade de São Paulo São Paulo SP Brazil -Hospital das Clínicas, Faculdade de Medicina da Universidade de São Paulo, São Paulo, SP, Brazil.; 17 Departamento de Imunizações Sociedade Brazileira de Pediatria São Paulo SP Brazil -Departamento de Imunizações, Sociedade Brazileira de Pediatria, São Paulo, SP, Brazil.; 18 Universidade Federal Fluminense Niterói RJ Brazil -Universidade Federal Fluminense, Niterói, RJ, Brazil; 19 Universidade Federal do Rio Grande do Sul Porto Alegre RS Brazil -Universidade Federal do Rio Grande do Sul, Porto Alegre, RS, Brazil.

## Abstract

•The negative impact of infectious diseases and their immunoprevention during the different stages of a woman’s life requires a broad approach including adolescence, adulthood, pregnancy and the postmenopausal phase.

•Immunization of pregnant women should be a priority for the protection of the maternal-fetal dyad, especially in regions with high rates of infections preventable by immunization.

•Brazil has one of the most comprehensive vaccination programs in the world – the National Immunization Program (*Programa Nacional de Imunizações*, PNI) – that serves all age groups: newborns, children, adolescents, adults, pregnant women and older adults, as well as groups with special needs, such as adolescents, pregnant and older adult women.

•However, vaccination coverage remains below ideal for all available vaccines, especially among adolescents and pregnant women, and Febrasgo is committed to collaborating with the PNI to combat vaccine hesitancy.

•The gynecologist/obstetrician is the reference physician for women, therefore the access to information and updates regarding all vaccines recommended for their patients is extremely important for this professional, aiming at the greatest possible protection.

•The objective of this Febrasgo Position Statement is to bring an update to women’s vaccination schedule, covering some vaccines that are available, including new approved vaccines and those in the commercialization phase.

•This work is a compilation of the First Febrasgo Scientific Immunization Forum held in the city of São Paulo in October 2023 with the objective to update recommendations for vaccines in use and new innovative vaccines soon to be available.

## Recommendations

The following recommendations were developed in the First Febrasgo Women’s Vaccination Forum:

- Febrasgo recommends the following dose schedule for HPV vaccines:
- Not previously vaccinated against HPV: 9-20 years => HPV4 (available on the public network) or HPV9 (in shared decision) => two doses six months apart (0-6 months), from 21 to 45 years of age: three doses of HPV9 (at 0-2-6 months).
- Febrasgo preferably recommends the HPV9 vaccine for all age groups based on a shared decision between the healthcare professional and the woman/family.
- Febrasgo recommends the 9V vaccine for those already previously vaccinated with a complete or incomplete schedule with the 4V and 2V vaccines, and who wish to extend protection against other types of HPV, in a schedule according to age group and in a shared decision between the health professional and the woman/family.
- For the transgender population and users of pre-exposure prophylaxis (PREP) or post-exposure prophylaxis (PEP), Febrasgo recommends a dose schedule according to age group on the same schedule.
- Febrasgo recommends the following vaccines for pregnant women:
-Trivalent influenza*: single dose, intramuscular, annual during seasonal periods, at any stage of the pregnancy period;
-Triple acellular bacterial, adult type * (diphtheria, tetanus and pertussis) – Tdap or Tdap-IPV:
-Adult double* (diphtheria and tetanus) – DT: Tdap is recommended in all pregnancies;
- Women not vaccinated during pregnancy should be vaccinated in the puerperal period as early as possible.
- If Tdap is unavailable, it can be replaced by Tdap-IPV. Prescription is at the physician’s discretion.
-Hepatitis B*: three doses at intervals of zero, two and six months for those not vaccinated or susceptible;
-COVID-19*: single dose of the variant in circulation, intramuscularly, at any stage of pregnancy, six months after the last dose administered;
-RSV: single dose, intramuscularly, between 24 and 36 weeks of pregnancy.
- Febrasgo recommends that pregnant women avoid vaccinating against yellow fever, unless there is an epidemiological emergency, in which case the physician must evaluate the benefits and risks. If women breastfeeding children under six months of age need to be vaccinated, they must stop breastfeeding for ten days after vaccination.
- For women over 60 years of age, Febrasgo recommends vaccination to prevent *S. pneumoniae* with PCV13 or PCV15 in a single dose schedule. For those who already have a dose of PCV13 or PCV15, it is recommended to take a dose of PPSV23 after two to six months, with a booster dose after five years.
- For women over 50 years of age and immunocompromised women over 18 years of age, Febrasgo recommends the inactivated recombinant vaccine for HZ.
- Febrasgo recommends the trivalent influenza vaccine to protect women at all stages of life.
- Febrasgo recommends the RSV adjuvanted vaccine for women over 60 years of age, especially for those in higher risk groups (with cardiopathy, pneumopathy, diabetics, nephropathy and liver disease).
- Febrasgo recommends the dengue vaccine for women aged four to 60 years in two doses, three months apart, subcutaneous application. It has the potential to protect women against this significant public health threat.
- Febrasgo recommends the ACWY meningococcal conjugate vaccine for those vaccinated in childhood, with a booster at 11 years of age or five years after the last dose. For unvaccinated people up to 15 years old, in two doses, five years apart, and from 16 years old, in one dose.
- For adolescents not vaccinated in childhood, Febrasgo recommends: meningococcal B vaccine in two doses, at least one month apart or six months apart with the vaccine authorized for that interval. These vaccines are not interchangeable.

## Background

### HPV vaccination

Persistent infection with oncogenic types of human papillomavirus (HPV) is a necessary antecedent condition for the occurrence of cervical cancer.^(1-3)^ In Brazil, a woman up to 45 years of age dies from cervical cancer at every 80 minutes.^(4)^ HPV infection is very prevalent, with a peak of infection occurring in adolescents and young adults and less frequently, although not less important, after the age of 45.^(3)^

The target age for HPV vaccination suggested by the World Health Organization (WHO) is between nine and 13 years old. From April 2024, the National Immunization Program (*Programa Nacional de Imunizações*, PNI) in Brazil makes the vaccine available for boys and girls aged nine-14 years on a single dose scheme.^(5,6)^

Over the last 10 years, several studies have shown significant evidence that one dose of the HPV vaccine can provide protection equal to two or three doses (depending on age) in areas with high vaccination coverage. Such results, added to the difficulties faced by many countries in incorporating vaccination against HPV, motivated the WHO in 2022, and the Pan American Health Organization (PAHO) in 2023, after thorough analysis by their technical-scientific committees (Strategic Advisory Group of Experts on Immunization [SAGE] and Technical Advisory Group [TAG]) to the position in favor of adopting a single-dose vaccination schedule of the HPV vaccine up to 20 years of age; two doses at six-month intervals from 21 years of age; and three doses for immunocompromised people, leaving it up to countries to adopt or not this recommendation.^(7)^

Thus, considering the recommendations of the WHO and PAHO with evidence showing that the single dose regimen of the HPV vaccine for people aged nine-20 years without immunosuppression can provide protection against cervical cancer equal to that of the two or three dose regimen, the difficulty of incorporating the vaccine in many countries and achieving adequate coverage in the second dose of the HPV vaccine, and the increase in vaccination coverage in countries where the single dose was adopted, the participants of the Technical Advisory Chamber of Immunizations (CTAI) of the Department of the National Immunization Program (DPNI) recommended and the Ministry of Health followed the recommendation, and started to adopt a single dose of the HPV vaccine in the PNI for adolescents aged nine-14 years. The single dose of HPV is adopted and the recommendations for other groups (immunosuppressed and victims of sexual violence) are maintained.^(6,7)^

For women with a history of treatment for cervical intraepithelial neoplasia grade 2 (CIN 2) or a more severe lesion, long-term follow-up data have shown that they are at increased risk of cancer for at least 25 years after treatment and the rate of persistent or recurrent disease varies from 4% to 18%.^(8)^

On the other hand, some authors have already published evidence that vaccination against HPV reduces the recurrence of high-grade lesions after surgical treatment, especially when related to HPV16 and HPV18.^(9)^ This evidence has been confirmed recently by more robust studies.

An important systematic review with meta-analysis published in 2020 showed that adjuvant HPV vaccination in the context of surgical excision for CIN 2 or more severe lesion is associated with a reduced risk of recurrent cervical dysplasia in general, and a reduction in risk of recurrent lesions caused by the most oncogenic strains (HPV16 and HPV18). This study concluded that vaccination against HPV should be considered in adjuvant treatment of patients undergoing surgical excision for CIN 2 or higher.^(10,11)^

This evidence led committees of medical societies and regulatory bodies, such as the American Society for Colposcopy and Cervical Pathology (ASCCP) and the Febrasgo Specialized National Commission (CNE) on Vaccines to publish new guidelines recommending vaccinating unvaccinated adult women or those with incomplete vaccination, in a scheme recommended according to age and up to the limit of 45 years of age. The timing of vaccination must be close to the therapeutic procedure, and can be started pre-, peri- or post-operatively.^(12,13)^

In relation to victims of sexual violence, studies suggest including this group in the vaccination against HPV, considering that individuals who have suffered sexual violence normally live in an environment where there is the possibility of recurrence. Such evidence has grown and consequently led to the publication of new guidelines by committees of medical societies and regulatory bodies, which recommend vaccination against HPV in these situations.^(14-16)^

In 2022, the WHO published their position and update regarding the HPV vaccine, recommending the consideration of the following population as a priority for HPV vaccination in public health programs: immunocompromised women and men, including those living with HIV, as well as children and adolescents who have suffered sexual abuse, due to the increased risk of contracting HPV-related diseases.^(17)^

National data show that up to 30% of victims of sexual violence later develop HPV lesions. A recent technical note published by the Ministry of Health included victims of sexual violence as a priority group for vaccination against HPV. It established that people aged nine-14 years should receive two doses of the vaccine at six-month intervals, and people aged 15-45 years, patients with HIV, solid organ transplants or bone marrow transplants and cancer, as well as immunosuppressed patients should receive three doses of the vaccine at intervals of zero, two and six months.^(18)^

The importance of vaccination against HPV in different risk groups was discussed in that presentation, providing data for the PNI, which currently covers the age group from nine to 45 years and seeks to balance sexes. During the presentation, the use of HPV vaccination was also discussed as a focus on preventing new infections with other HPV subtypes and reducing the recurrence of lesions in individuals already exposed to the virus, especially in women previously treated for high-grade CIN. According to the representatives at the forum, a consensus was reached that the vaccine should be administered even to patients who have already been infected, taking into account the protection that can be offered to other subtypes of the HPV virus, as well as improving immunity of the patient for the subtype already acquired. Studies have shown reductions of more than 70% in the recurrence of lesions in vaccinated patients undergoing high-grade lesion treatment procedures. The benefits of vaccination for reducing the recurrence of lesions in sites other than the cervix, such as the anus and larynx, were also highlighted. The importance of considering and including the LGBTQ+ population as a group in vaccination programs, as well as people with reduced access to health services, was emphasized. Vaccination was also suggested for users of pre-exposure prophylaxis (PREP) and post-exposure prophylaxis (PEP) aged 18 and over.^(19)^

### Vaccination against respiratory syncytial virus (RSV)

The RSV was isolated in 1955 and is spread through respiratory secretion. Clinical symptoms vary according to age.^(20)^ The burden of the disease tends to be underestimated in older adults. The most serious forms affect the extremes of age – babies and older adults –, and practically 100% of children become infected up to the age of two years. More than 95% of lower respiratory infections and 97% of deaths attributed to the RSV occurred in low- and middle-income countries.^(21)^

Bronchiolitis generally develops in young children, recurrent wheezing in older children, the common cold in healthy adults and asthma exacerbation, chronic obstructive pulmonary disease and severe respiratory disease in older adults. The strategy of actively vaccinating pregnant women was discussed with the aim of achieving the transfer of protective antibodies to the fetus and consequently to newborns. Passive immunization with two currently licensed monoclonal antibodies – nirsevimab and palivizumab – was also shown to protect newborns.

There are also two RSV vaccines approved for older adults: the adjuvanted vaccine and the non-adjuvanted vaccine, both using the F protein in its pre-fusional conformation.^(22-24)^

Regarding the prevention of the disease in young infants, the different passive immunization strategies were discussed, comparing maternal vaccination and immunization using monoclonal antibodies directly administered to the newborn.

In clinical studies with a candidate vaccine for pregnant women, an imbalance was observed between those vaccinated and the placebo group, and an increased risk of premature birth in the vaccinated group led to the interruption of this clinical trial.^(25)^

The currently approved vaccine, also with the F protein, pre-fusional, bivalent, against RSV genotypes A and B, non-adjuvanted, did not present a significant incidence of premature births, and it is the only one in the world indicated for pregnant women. The effectiveness of this vaccine for preventing severe lower respiratory tract disease in infants was considered excellent; 81.8% in the first three months, decreasing to 69.4% after six months. For the mild form – lower respiratory tract infection without hospitalization – the effectiveness was lower, 57.1% in the first trimester and 51% in the first six months of life, reaching the primary objective. In the secondary objective after 12 months, protection of up to 45% prevention of RSV infections was still maintained, and after 360 days, statistical criteria with a significant confidence interval were also reached.^(26)^

The seasonality of the virus and gestational age must be considered to define the ideal time to apply the vaccine during pregnancy and induce enhanced transfer of antibodies. The studies were conducted with administration of the vaccine at weeks 24-36 of pregnancy. The Advisory Committee on Immunization (ACIP) recommends administering the vaccine between 32 and 36 weeks, noting that during this interval there is a reduction in the risk of serious adverse events, such as prematurity and disorders such as hypertension in pregnancy, including preeclampsia.^(22)^

The non-adjuvanted vaccine is authorized for pregnant women and registered by Anvisa. It must be applied between the second and third trimesters (weeks 24-36) to protect newborns. During clinical studies, some adverse effects were observed when administering the vaccine. The most common were pain at the vaccination site (40%), headache (31%) and muscle pain (26%). In clinical trials, low birth weight (5.1% non-adjuvanted vaccine versus 4.4% placebo) and neonatal jaundice (7.2% non-adjuvanted vaccine versus 6.7% placebo) were also observed in newborns followed in the studies.^(26)^

Passive immunization with the monoclonal antibody nirsevimab administered to newborns and infants under one year of age is considered an excellent alternative to the vaccine during pregnancy, providing high protection to the baby and not posing a risk to the pregnancy. However, industrial-scale production is still more expensive and complex, which makes universal coverage difficult.^(26,27)^

The RSV can cause severe diseases in older adults, especially respiratory complications. Since there is no specific treatment, vaccination is the best form of prevention. Although this respiratory infection is less frequent than influenza and COVID, RSV was more associated with severe diseases among hospitalized older adults.^(28)^

Two vaccine formulations against RSV are being launched for use in older adults, both using the F protein in their pre-fusional conformation as an antigen. Another vaccine with messenger RNA technology is in phase 3 and has not yet been approved.

In Brazil, RSV vaccines from two laboratories were approved by the National Health Surveillance Agency (Anvisa) for use throughout the national territory. These vaccines protect people over the age of 60 against RSV and diseases associated with the virus, such as severe acute respiratory syndrome and pneumonia. The vaccines are currently in the registration phase with the Drug Market Regulation Chamber (CMED) and should be released for sale soon, initially in the private vaccination network.^(29,30)^

In both RSV vaccines studies in older adults, some rare cardiac (atrial fibrillation) and neurological (Guillain-Barré syndrome) events occurred, but the safety profile was considered acceptable. Post-marketing studies for both vaccines will be maintained to evaluate effectiveness and safety.^(30,31)^

Both vaccines have demonstrated moderate to high efficacy in preventing RSV disease, and surveillance agencies suggest that vaccination should prevent considerable morbidity in these patients over 60 years of age. To date, there is no evidence if there will be a need for revaccination. It was also observed that the co-administration of the RSV vaccine with the influenza vaccine did not demonstrate interference in the immune response of both, with an adequate safety profile.^(32)^

### Vaccination against pertussis, tetanus and diphtheria in pregnant women

An increase in the occurrence of pertussis cases was observed in several countries from 2010 onwards, even with high vaccination coverage. In 2012, 41,880 cases of pertussis were recorded in the USA; 2,746 cases in children under one year of age, and only 2.6% of pregnant women were immunized during pregnancy.^(23)^ A significant increase in the incidence of pertussis cases was observed between 2011 (2,248 cases ) and 2014 (8,614 cases) in Brazil, with 61% diagnosed in children under one year of age and the majority of this group under the age of two months, with worsened outcomes.^(33)^

In 2014, Brazil’s PNI included the adult-type triple acellular vaccine (Tdap) for pregnant women between 27 and 36 weeks of pregnancy. In 2017, the recommendation was changed to administer the vaccine from the week 20 onwards with the aim of protecting newborns through passive immunization via the placenta. The Tdap vaccine is considered effective and safe.^(33)^

According to the *Advisory Committee for Immunization Practices* (ACIP, USA) and the *Center of Diseases Control* (CDC, USA), when reviewing data from the *Vaccine Adverse Reporting System* (VAERS) surveillance system, as well as in smaller studies, no elevated risks or unusual patterns of adverse effects were found. The safety of Tdap vaccines in pregnant women was also found in a study in Brazil.^(34)^

The Tdap vaccine is recommended for pregnant women after 20 weeks of pregnancy and also for family members and cohabitants in order to protect the young infant (cocoon strategy). The Tdap vaccine administered during pregnancy, in addition to presenting a 90% reduction in the risk of children contracting whooping cough in the first months of life, provides additional protection of around 70% throughout the first year of life.^(35)^

### Influenza and COVID-19 vaccine in pregnant women

Flu vaccination is highly recommended for pregnant women due to the increased risk of symptoms and complications during pregnancy. Furthermore, by vaccinating pregnant women, the fetus and the newborn in the first six months of life are protected through the passage of antibodies via the placenta and lactation.^(36)^

There are two vaccine formulations available: trivalent (public system) and tetravalent (private clinics). These vaccines are considered safe, have minimal local adverse events, and are recommended annually as the immunity lasts six-12 months. Pregnant women and puerperal women up to the first 45 days are considered risk groups and should be given priority for vaccination. Vaccination significantly reduces the risk of gestational complications and infections in newborns.^(37)^

With the emergence of the COVID-19 pandemic, the vaccination situation against the causative agent SARS-CoV-2 has evolved since mid-2020, with regular updates from the Ministry of Health regarding morbidity and vaccination coverage. Four types of vaccines are currently authorized in Brazil: viral vector vaccine, inactivated vaccine and modified RNA vaccines (mRNA). The Ministry of Health does not recommend the viral vector platform vaccines for pregnant and puerperal women, but other vaccines are permitted. Inactivated or modified RNA (mRNA) COVID-19 vaccines are considered safe for pregnant and puerperal women, with no risk of malformations to the fetus and with the recommendation of administration throughout pregnancy and puerperal period up to 45 days after birth. The mRNA vaccine is currently recommended for pregnant women, with doses and formulations varying according to age group. The vaccination schedule for COVID-19 in pregnant women follows the recommendation for adults, which involves two doses eight weeks apart. Pregnant and puerperal women are considered risk groups for both influenza and COVID-19.^(38)^

### Vaccination against *Streptococcus pneumoniae*

*Streptococcus pneumoniae* is the leading cause of pneumonia, meningitis and bacteremia worldwide. There are more than 90 identified serotypes of the bacteria and the serotypes are not equally pathogenic. Antibiotic resistance is a cause for global concern.^(39)^

The vaccines developed against *S. pneumoniae* are formulated with capsular polysaccharide and can protect against different serotypes, thereby reducing the outcomes of invasive pneumococcal disease. Children are the main carriers of pneumococci and by immunizing them with conjugate vaccines, the circulation of the serotypes contained in the vaccines will be eliminated. By reducing colonization, these children stop transmitting it to cohabitant adults, generating an effect known as indirect protection. This brings the possibility to prevent more cases in adults and older adults than through the direct effect of vaccination in older adults.^(39)^

Approximately 140 countries use the 13-valent pneumococcal conjugate vaccine (PCV13), and Brazil still uses the 10-valent vaccine (PCV10) in the PNI. A significant reduction in registrations of vaccine serotypes of the PCV10 was observed in recent years.^(40)^

Serotype 19A currently accounts for 53% of cases of invasive disease in children. This serotype, together with serotype 3 and serotype 6A is responsible for 70% of cases of invasive disease at all ages. These three serotypes were added in the PCV13, compared to the PCV10. The serotype 19A, currently circulating in Brazil and several other countries, belongs to a clonal complex associated with resistance to penicillin (72%) and ceftriaxone.^(40)^

The PCV10 is currently available through the PNI. The PCV13 is available at the Reference Centers for Special Immunobiologicals (*Centro de Referência para Imunobiológicos Especiais*, CRIE) and incorporates the three extra serotypes. Even though the 15-valent vaccine (PCV15) was licensed recently, the two additional 13-valent serotypes (22F and 33F) represent only 3% of cases registered in Brazil.^(39,40)^ The 20-valent (PCV20) and 21-valent (PCV21) conjugate vaccines, still in the approval phase, also exist. The 23-valent pneumococcal polysaccharide vaccine (PPSV23) should only be used in special situations and in older adults. In the USA, for at-risk adults over 19 years of age, vaccination with PCV15 is recommended followed by the PPSV23 in a sequential schedule two months apart, or just one dose of PCV20.^(40,41)^

The forum’s current recommendation for the national scenario is to complete older adult women’s vaccination schedule with PCV13 or PCV15. For those who have already taken a dose of PCV13 or PCV15, a dose of PPSV23 is recommended for older adult women and risk groups, with a booster dose after five years.

For those who have already received a dose of PPSV23, a one-year interval is recommended for the application of PCV13 or PCV15. The second dose of PPSV23 must be given five years after the first, maintaining six-12 month intervals with the PCV13 or PCV15. For those who have already received two doses of PPSV23, a dose of PCV13 or PCV15 is recommended at a minimum interval of one year after the last dose of PPSV23. If the second dose of PPSV23 was administered before the age of 60 years, a third dose is recommended after that age at a minimum interval of five years since the last dose.

### Herpes zoster (HZ) vaccination

The vaccine against the virus that causes HZ currently in use is the recombinant vaccine. Since this is a recombinant, inactivated vaccine, it is authorized for immunocompromised patients. The live attenuated vaccine has been withdrawn from the market and is no longer part of the updated recommendations.^(1)^

All societies recommend to start vaccination against HZ from the age of 50 in a two-dose schedule at two-month intervals. The recombinant vaccine can generate high levels of antibodies, and follow-up studies have shown high efficacy after nine and a half years of administration of the complete two-dose regimen.^(41)^

Findings in a recent study demonstrated an efficacy of 91% in preventing acute episodes of HZ in all age groups, with an upper limit of up to 89 years.^(42)^

Therefore, the recommendation to vaccinate all women over 50 years of age was established, as stated in the recombinant vaccine leaflet. As current data show an increased occurrence of cases of the disease in people over 80 years of age, it was a consensus not to establish age limits for vaccination, but to evaluate and recommend vaccination in each specific case.

Considering chickenpox infection, if the patient is over 50 years old and has no history of chickenpox, with negative IgG, vaccination with the recombinant vaccine should be recommended.

Regarding cases of immunosuppression, vaccination against HZ is recommended from 18 years of age. In individuals who have already had HZ, the Brazilian Society of Immunizations (SBIm) ideally recommends the vaccination from six months after infection. However, a study from Germany recommends vaccination starting two to three months after infection, and the CDC and the United Kingdom recommend vaccination after the acute phase of the disease has resolved.^(43)^

The recurrence rate of HZ is approximately 6% in immunocompetent individuals, and recurrence is more frequent in women and younger patients.^(44)^

### Influenza vaccination in adult women

Influenza is an acute respiratory disease caused by influenza A and B viruses. It occurs in the form of an epidemic, mainly in winter, with the particularity that viruses modify their antigenic characteristics with great frequency, making annual vaccination against influenza an important public health measure. When the influenza virus affects immunocompromised individuals and older adults over 60 years of age, the risk of complications increases, making it the second leading cause of death in this group of patients.^(45)^

In Brazil, as a condition of technology transfer, the trivalent vaccine, which contains two strains of influenza A (one H1N1 and the other H3N2) and one of influenza B, has been used in annual seasonal programs. There are two strains of influenza B virus (Victoria and Yamagata), and the latter is currently considered to have disappeared from circulation. Starting in 2021, with the aim of improving the immune response to the influenza virus in vulnerable populations, vaccines with adjuvants and a higher concentration of antigens were developed.^(46)^

In Brazil, from 2023, began the recommendations for the high-dose vaccine, which has four times the dose of the traditional ones, i.e., 60 µg of antigens instead of 15 µg. These vaccines showed greater protection for older adults (around 20%), especially for the H3N2 strain. The vaccine generates a peak of immunity after four or six weeks, and given the decrease observed after the third month, seasonal vaccination is extremely important.^(28)^

In a meta-analysis study of adults over 65 years of age, the quadrivalent vaccine reduced the incidence from 6% to 2.4% in eight trials and in another study, it decreased from 2.3% to 0.9%. Studies of patients with cardiovascular diseases also showed benefits in relation to mortality.^(47)^ The strategy adopted according to the circulation of the strains will be to abandon the use of the quadrivalent vaccine and reinforce the trivalent vaccine for a greater adequacy.

Dengue vaccination

The dengue virus belongs to the Flaviviridae family and is composed of four distinct, closely related serotypes (DENV1 - DENV4). All four serotypes can cause the disease spectrum.^(48)^

Dengue represents a significant public health problem, with high prevalence in several regions, including Brazil. In 2019, the WHO classified dengue as one of the ten main threats to global health.^(49,50)^

The “first generation” of the dengue vaccine was not widely adopted due to concerns about adverse events and the difficult to adopt dosage schedule. However, the second generation of the vaccine, TAK-003, did not present associated safety problems in studies and at the moment, many efforts are focused on demystifying the lack of safety attributed to the old vaccine.^(51)^

In Brazil, the number of dengue cases in adults over 30 years of age has increased, as well as in those over 60 years of age, with a significant proportion also in adolescents and young adults. Severe cases have increased among older adults, and the predominant mortality is observed above 40 years of age.^(49)^

Chronic conditions such as hypertension, diabetes, and kidney and cardiovascular disease increase the risk of progression to severe dengue. Diabetes increases the risk of progression to severe dengue by 4.3 times, and kidney disease is associated with an 11 times greater risk of death.^(50)^

The new vaccine TAK-003 differs from the first generation due to its genetic base that uses the serotype 2 virus and contains strains of all four serotypes, as well as structural and non-structural proteins to stimulate a broader immune response. The phase 3 DEN-301 study involved more than 20,000 children and adolescents, and evaluated the vaccine’s effectiveness over four and a half years. The results showed effectiveness in preventing hospitalizations and severe forms of dengue, especially among individuals previously exposed to the virus.^(52)^

This vaccine was effective against all serotypes, with efficacy ranging from 48.9% to 95.1% depending on the serotype. Efficacy was significant for both seropositive and seronegative individuals. Although there was no protection for seronegative subjects for serotype 3 and type 4, protection could not be demonstrated in the study, as there was no circulation of this serotype during the study period.^(53)^

Studies are evaluating the concomitant use of the TAK-003 vaccine with yellow fever, hepatitis A, and HPV vaccines. Preliminary results suggest that their coadministration does not negatively affect the immunogenicity of the vaccines.^(53)^

The vaccine was approved in Brazil for individuals aged four to 60 years. Since this is a live attenuated virus vaccine, it is contraindicated in pregnant, lactating and immunocompromised women.^(52)^

### Vaccination against meningitis

The meningococcal disease was first described in Switzerland in 1800 and there were epidemics of meningitis among young conscripts during the world wars, leading to the introduction of chemoprophylaxis and later, vaccination.^(1)^

Routine vaccination against meningitis was introduced in Brazil in 2010 in PNIs and contributed significantly to the reduction in cases of meningococcal diseases.^(54)^

The extreme importance of vaccinating adolescents is well known, as most asymptomatic carriers are in this age group. In 2017, routine vaccination against serogroup C was implemented in adolescents, and replaced by the tetravalent ACWY serogroup vaccine in 2020. The MenC vaccine was the first one approved against meningococcus, and its introduction into public programs in several countries around the world showed a marked reduction in the number of cases of the disease among those vaccinated.^(1)^

In CRIE, the MenC vaccine is available to: people who use immunosuppressive drugs (two doses, eight weeks apart and a booster dose in five years); patients undergoing cancer treatment (two doses eight weeks apart and a booster dose in five years); patients with cerebrospinal fluid leak (one dose with a booster dose in five years); patients with cochlear implants (one dose with a booster dose in five years); patients with trisomies (one dose); patients with deposition diseases (one dose); patients with chronic liver disease and disabling neurological disease (one dose).^(55)^

Adherence to the vaccine during the period evaluated was higher among infants and young children, with a median of 91.0% (confidence interval [CI]: 87%-98%) at the age of three-five months in the period from 2011 to 2021, and of 84.5% (CI: 77.2%-88.5%) at 12-15 months of age in the period from 2012 to 2021.^(54)^

The vaccine against serogroup B of meningococcal disease is not included in the PNI, and two formulations are available in the private network; one vaccine can be applied from two months to 50 years of age and the other from 10 to 25 years of age.^(54)^

Febrasgo recommends vaccination against meningococcus B for all adolescents up to 20 years of age routinely and for immunosuppressed women of any age.

In relation to pregnancy and lactation, meningococcal vaccines should be postponed due to the lack of data guaranteeing the safety of vaccines in this group, with the exception of outbreak situations.^(1)^

After the introduction of the conjugate vaccine against ACWY serogroups in Brazil, the total incidence coefficient of meningococcal disease reduced from 1.5 cases to 0.4 cases per 100 thousand inhabitants comparing the period before (2007-2010) and after vaccination (2017-2020). This vaccination has been recommended by scientific societies preferably in the calendar for children and adolescents, expanding the spectrum of protection in relation to the monovalent C vaccine. In the period from 2007 to 2020, the most frequent serogroups in Brazil were C (8,811 cases), B (2,662), W (815 cases) and Y (215 cases).^(54)^

The ACWY meningococcal conjugate vaccine contains polysaccharides from the four main serogroups, expanding the spectrum of protection, and is currently recommended by all medical societies. Vaccination with the ACWY conjugate vaccine is recommended for risk groups, as well as travelers visiting regions with a high incidence of the disease, such as the meningitis belt (Africa), in which it should be administered seven to 10 days before travelling and consequently, the possible exposure.^(1)^

In a recent retrospective observational study, the four-component protein vaccine against meningococcus B also provided indirect protection against other non-B serogroups, as it contains antigens shared with other serogroups. Furthermore, cross-protection of 4CMenB against *N. gonorrhoeae* was demonstrated, supporting the potential of vaccination strategies to prevent gonorrhea. However, new follow-up studies are needed to assess this possible cross-protection.^(56)^

Recommendations and vaccination schedules vary according to age, risk group and type of vaccine, and routine immunization and vaccination in specific epidemiological situations are very important.^(55)^

The Food and Drug Administration (FDA) approved the ACWYB vaccine formulation while the forum was held.

The National Specialized Commission on Vaccines of Febrasgo recommends the vaccination with ACWY conjugate vaccines for all pre-adolescents and adolescents, with a single dose and a booster dose after five years. Whenever possible, adolescents and young adults should also receive the meningococcal B vaccine in a two-dose schedule at one-two month intervals. The National Specialized Commission on Vaccines emphasizes that multivalent vaccines (ACWY) and those against meningococcal B are indicated for all adolescents and young people up to 20 years of age and for women in the risk group for meningococcal disease.^(55)^

### Yellow fever vaccination

The yellow fever vaccine was developed in 1929. It is one of the oldest vaccines and essential for controlling the disease in endemic regions. Yellow fever is transmitted mainly in wild areas and is a notifiable disease.^(57)^ Since 1942, there have been no recorded cases of yellow fever in urban areas in Brazil and two vaccines are available globally, most of which are produced in Brazil. All yellow fever vaccines use the attenuated virus strain 17D, with variations 17DD and 17D-204. The immune response after vaccination is strong and long-lasting, with 97.5% seroconversion in vaccinated adults. In a study of children under two years of age, a lower level of antibodies to MMR vaccines was observed when administered in conjunction with the yellow fever vaccine. Therefore, the MMR vaccine should not be applied together with the yellow fever vaccine, and the 30-day interval must be respected.^(58)^

Since 2017, the WHO has recommended that a single dose of the vaccine is sufficient for immunization. The most common side effects are pain and erythema at the site, fever and myalgia, and serious allergic reactions are rare. The yellow fever vaccine is recommended from the age of nine months throughout Brazil and for travelers heading to endemic areas. Note that vaccination is required at least 10 days before the travelling date when visiting endemic areas. Some countries require an International Certificate of Vaccination and Prophylaxis (ICVP) with registration of the yellow fever vaccine, provided for by the International Health Regulations (IHR).^(1)^

The Brazilian Ministry of Health recommends vaccination at nine months and a booster at four years. People who receive the split vaccine are considered immunized during eight years at least.^(1)^

The vaccine is contraindicated for children under six months of age, pregnant women, women breastfeeding infants under six months of age and severely immunocompromised patients, among other conditions. Vaccination in people over 60 years of age must be analyzed individually, considering risks and benefits.^(1)^

Febrasgo recommends that pregnant women avoid vaccination, unless there is an epidemiological emergency, in which case the physician must evaluate the benefits and risks. If women breastfeeding children under six months of age need to be vaccinated, they must stop breastfeeding for 10 days after vaccination.

**What is the current PNI recommendation, dose and indications, on the HPV vaccine available on the public network?**

Following the recent Technical Note from the Ministry of Health, the HPV4 vaccine – the only one available in the public network to date – is now offered by the PNI, covering girls and boys aged nine to 14 years: one dose in Basic Health Units (UBS). In a temporary rescue strategy for those who missed the opportunity at the recommended age, the PNI is making one dose available to teenagers aged 15-19 years who have never been vaccinated at the UBS. The recommendation for immunocompromised people continues to be the vaccine in three doses for people aged nine to 45 years in the CRIE. The PNI also included people aged nine to 45 years who were victims of sexual abuse, unvaccinated or incompletely vaccinated, in the scheme provided for in the leaflet for the age group (two doses from nine to 14 years, or three doses from 15-45 years). Vaccination can be carried out in units that care for victims of abuse or in UBS with referral by the professional.

**How to take advantage of the opportunity in a routine consultation and recommend the RSV vaccine?**

The new vaccination strategies available for pregnant women and women aged over 60 years can be addressed. During antenatal care and for women over 60 years of age, there are opportunities to show the importance of vaccination in preventing diseases that impact the baby, as well as the complete safety of the vaccine to prevent RSV. Although it has a seasonality from May that can extend until September, a single dose can be taken at any time of the year. The licensed RSV adjuvanted vaccine provides adults over 60 years of age with protection for up to two seasons, which allows the vaccine to be indicated at any time of the year in a single, intramuscular dose to protect against RSV infection and the risk of its complications, such as pneumonia, or decompensation of pre-existing comorbidities, such as diabetes, heart disease and others. This vaccine is especially recommended for older adults in higher risk groups (with cardiopathy, pneumopathy, diabetes, nephropathy and liver disease).

**How to recommend the RSV vaccine to pregnant women?**

The only vaccine licensed for pregnant women is non-adjuvanted and can be used to prevent lower respiratory tract disease and severe lower respiratory tract disease caused by the RSV in children from birth to six months of age through the active immunization of pregnant women in the second or third trimester of pregnancy (24-36 weeks). This vaccine is administered in a single dose, intramuscularly, and should not be administered together with other vaccines, for example, pertussis, and a two-week interval between applications must be respected.

The RSV adjuvanted vaccine for RSV is authorized for use in older adults and should not be administered to pregnant women. The RSV non-adjuvanted vaccine is recommended for pregnant women and older adults.

**Which vaccines are recommended during pregnancy and the puerperal period?**

Vaccines for influenza, diphtheria, tetanus and pertussis (DT + Tdap), hepatitis B, COVID-19 and RSV are recommended during pregnancy and the puerperal period.

**When should the pertussis vaccine be recommended? Is it necessary to vaccinate contacts?**

The Tdap vaccine is recommended in one dose for pregnant women after 20 weeks of pregnancy and also for family members and cohabitants in order to protect the young infant (cocoon strategy). It is available in the PNI.

**What is the recommended dosage of meningococcal B and ACWY vaccines for adolescents aged nine years and over?**

Febrasgo recommends one dose of the MenACWY vaccine and two doses of meningitis B vaccine at 30-day intervals between doses for adolescents and young adults. Remember that these vaccines can be administered on the same day as others such as the HPV vaccine. In addition, as vaccines against serogroup B meningitis are recent, most adolescents and young adults have not yet been immunized and represent an important focus of asymptomatic carriers given the typical behavior of this age group, such as taking part in crowds and group activities.

**When to indicate and what are the schedules and recommendations for pneumococcal vaccines in older adult women?**

The vaccination against pneumonia is recommended in routine consultations for older adult women. The PCV13 or PCV15 and PPSV23 are indicated in the following scheme: starting with a dose of PCV13 or PCV15, followed by a dose of PPSV23 after six to 12 months, and a second dose of PPSV23 five years after the first. For those who have already received a dose of PPSV23, a one-year interval is recommended for the application of PCV13 or PCV15. The second dose of PPSV23 must be given five years after the first, maintaining six to 12-month intervals with the PCV13 or PCV15. For those who have already received two doses of PPSV23, a dose of PCV13 or PCV15 is recommended at a minimum interval of one year after the last dose of PPSV23. If the second dose of PPSV23 was administered before the age of 60 years, a third dose is recommended after that age at a minimum interval of five years since the last dose. These vaccines are available in the private network. At the UBS, the PPSV23 is available free of charge only to those in long-term care institutions for older adults and high-risk groups.

**When to indicate the HZ vaccine in older adult women and what are the schedules and recommendations?**

If the woman has not been vaccinated at age 50, the vaccine is indicated at any time, since it is routinely recommended from age 50 onwards in the following schedule: recombinant inactivated vaccine (RZV) in two doses, two months apart (0-2). Vaccination is recommended even for those who have already developed the disease after the condition has resolved. For those previously vaccinated with the attenuated vaccine (LZV), RZV is recommended respecting the minimum two-month interval between them. In immunocompromised patients, RZV is recommended from 18 years of age onwards.

**What is the effectiveness and indication of the new tetravalent dengue vaccine approved by Anvisa?**

The new dengue vaccine is effective against all dengue serotypes, as well as in individuals who have been infected by a serotype in the past. The vaccine protects those who have never been infected, and those who have already had dengue and are at greater risk of having severe forms. After completing the vaccination schedule, the overall effectiveness of the new tetravalent dengue vaccine against infection is 60%-80%, while the effectiveness against severe forms is 85%-90%. The vaccine must be administered in a two-dose schedule at three-month intervals. There is no recommendation for booster doses.

## Final considerations

In Brazil, the PNI currently makes 49 immunizations available free of charge to the population in more than 38 thousand vaccination rooms across the country, a number that can double in specific campaigns. There are also 52 CRIE spread across the country to offer vaccines for populations with special needs. A variety of national immunization schedules suit the different age groups and conditions, such as children, adolescents, adults, older adults, pregnant women and especially those with comorbidities, chronic diseases or immunosuppressive states. Brazil’s vaccination record began to improve significantly in the 1980s, when infectious diseases were more visible and vaccination was robustly adopted, and vaccination coverage increased, reaching highs in the 2000s. However, since 2015-2016, there has been a drop in vaccination coverage, worsened by the COVID-19 pandemic. This has been partially attributed to a generation of individuals who have not witnessed large-scale infectious diseases and therefore, have lower risk perception given their lack of disease knowledge. Current challenges include improving vaccination coverage data records to obtain reliable data, improving access to vaccines with greater availability of vaccination times and locations, in addition to combating vaccine hesitancy through effective and clear communication about the benefits and possible risks. Coverage for vaccines such as Tdap in pregnant women and HPV in adolescents is low, with the HPV vaccine showing particularly low rates for the second dose, especially in boys. Vaccine coverage for yellow fever increased significantly after an epidemic in 2017, showing the influence of risk perception on vaccination adherence. Therefore, different strategies and measures are needed to improve vaccination coverage and they must be emphasized in different regions, such as giving necessary attention to communication at the national level, adjustments in information systems, simplification of vaccination calendars and microplanning by states. Multi-vaccination and communication actions targeting specific vaccines are suggested to increase coverage, and the forum presentation concludes that support and partnership from societies, scientific communities and healthcare professionals are vital to the success of vaccination strategies, especially in a country with great cultural and social diversity like Brazil.

## References

[1] Federação Brasileira das Associações de Ginecologia e Obstetrícia (2021). Programa vacinal para mulheres.

[2] Ferlay J, Ervik M, Lam F, Laversanne M, Colombet M, Mery L (2020). Global Cancer Observatory: Cancer Today.

[3] Bruni L, Albero G, Serrano B, Mena M, Collado JJ, Gómez D (2023). Human papillomavirus and related diseases in United States of America.

[4] Ministério da Saúde, Instituto Nacional de Câncer (2021). Atlas de Mortalidade.

[5] Markowitz LE, Dunne EF, Saraiya M, Chesson HW, Curtis CR, Gee J (2014). Human papillomavirus vaccination: recommendations of the Advisory Committee on Immunization Practices (ACIP). MMWR Recomm Rep.

[6] Ministério da Saúde, Secretaria de Vigilância em Saúde e Ambiente, Departamento do Programa Nacional de Imunizações, Coordenação-Geral de Incorporação Científica e Imunização (2024). Nota Técnica No. 41/2024-CGICI/DPNI/SVSA/MS: atualização das recomendações da vacinação contra HPV no Brasil.

[7] World Health Organization (2024). Cervical cancer.

[8] Kalliala I, Athanasiou A, Veroniki AA, Salanti G, Efthimiou O, Raftis N (2020). Incidence and mortality from cervical cancer and other malignancies after treatment of cervical intraepithelial neoplasia: a systematic review and meta-analysis of the literature. Ann Oncol.

[9] Joura EA, Garland SM, Paavonen J, Ferris DG, Perez G, Ault KA (2012). Effect of the human papillomavirus (HPV) quadrivalent vaccine in a subgroup of women with cervical and vulvar disease: retrospective pooled analysis of trial data. BMJ.

[10] Lichter K, Krause D, Xu J, Tsai SH, Hage C, Weston E (2020). Adjuvant human papillomavirus vaccine to reduce recurrent cervical dysplasia in unvaccinated women: a systematic review and meta-analysis. Obstet Gynecol.

[11] Jentschke M, Kampers J, Becker J, Sibbertsen P, Hillemanns P (2020). Prophylactic HPV vaccination after conization: a systematic review and meta-analysis. Vaccine.

[12] Roteli-Martins CM, Magno V, Santos AL, Teixeira JC, Nilma AN, Fialho SC (2022). Human papillomavirus vaccination for adult women. Rev Bras Ginecol Obstet.

[13] Sharpless KE, Marcus JZ, Kuroki LM, Wiser AL, Flowers L (2023). ASCCP Committee Opinion: adjuvant human papillomavirus vaccine for patients undergoing treatment for cervical intraepithelial neoplasia. J Low Genit Tract Dis.

[14] Seña AC, Hsu KK, Kellogg N, Girardet R, Christian CW, Linden J (2015). Sexual assault and sexually transmitted infections in adults, adolescents, and children. Clin Infect Dis.

[15] (2020). Human papillomavirus vaccination: ACOG Committee Opinion Summary, Number 809. Obstet Gynecol.

[16] Workowski KA, Bachmann LH, Chan PA, Johnston CM, Muzny CA, Park I (2021). Sexually transmitted infections treatment guidelines, 2021. MMWR Recomm Rep.

[17] World Health Organization (2022). Human papillomavirus vaccines: WHO position paper. Wkly Epidemiol Rec.

[18] Ministério da Saúde, Secretaria de Vigilância em Saúde e Ambiente, Departamento de Imunização e Doenças Imunopreviníveis, Coordenação-Geral de Incorporação Científica e Imunização (2023). Nota Técnica No. 63/2023-CGICI/DPNI/SVSA/MS. Inclusão de vítimas de violência sexual como grupo prioritário para vacinação contra o HPV, para pessoas de nove a 45 anos de idade, ainda não vacinados contra HPV.

[19] Wiley DJ, Monsonego EV, Lu S, Heather LS, Salem B, Giuliano AR (2012). Behavioral and sociodemographic risk factors for serological and DNA evidence of HPV 6, 11, 16, 18 infections. Cancer Epidemiol.

[20] Wu P, Hartert TV (2011). Evidence for a causal relationship between respiratory syncytial virus infection and asthma. Expert Rev Anti Infect Ther.

[21] Lozano R, Naghavi M, Foreman K, Lim S, Shibuya K, Aboyans V (2012). Global and regional mortality from 235 causes of death for 20 age groups in 1990 and 2010: a systematic analysis for the Global Burden of Disease Study 2010. Lancet.

[22] Kampmann B, Madhi SA, Munjal I, Simões EA, Pahud BA, Llapur C (2023). Bivalent prefusion F vaccine in pregnancy to prevent RSV illness in infants. N Engl J Med.

[23] Walsh EE, Pérez Marc G, Zareba AM, Falsey AR, Jiang Q, Patton M (2023). Efficacy and safety of a bivalent RSV prefusion F vaccine in older adults. N Engl J Med.

[24] FDA approves first Respiratory Syncytial Virus (RSV) vaccine Arexvy (2023). approved for individuals 60 years of age and older.

[25] Mejias A, Rodríguez-Fernández R, Oliva A, Peeples ME, Ramilo O (2020). The journey to a respiratory syncytial virus vaccine. Ann Allergy Immunol.

[26] Mazur NI, Testappen J, Baral R, Bardají A, Beutels P, Buchholz UJ (2023). Respiratory syncytial virus prevention within reach: the vaccine and monoclonal antibody landscape. Lancet Infect Dis.

[27] Esposito S, Abu Raya B, Baraldi E, Flanagan K, Martinon Torres F, Tsolia M (2022). RSV prevention in all infants: which is the most preferable strategy?. Front Immunol.

[28] Ministério da Saúde, Agência Nacional de Vigilância Sanitária (2023). Beyfortus® (nirsevimabe): novo registro.

[29] Surie D, Yuengling KA, DeCuir J, Zhu Y, Gaglani M, Ginde AA (2023). Disease severity of respiratory syncytial virus compared with covid-19 and influenza among hospitalized adults aged =60 years - IVY Network, 20 U.S. States, February 2022-May 2023. MMWR Morb Mortal Wkly Rep.

[30] Wilson E, Goswami J, Baqui AH, Doreski PA, Perez-Marc G, Zaman K (2023). Efficacy and safety of an mRNA-Based RSV PreF vaccine in older adults. N Engl J Med.

[31] Ministério da Saúde (2023). Agência Nacional de Vigilância Sanitária. Anvisa aprova registro de primeira vacina para bronquiolite.

[32] Papi A, Ison MG, Langley JM, Lee DG, Leroux-Roels I, Martinon-Torres F (2023). Respiratory syncytial virus prefusion F protein vaccine in older adults. N Engl J Med.

[33] Bridges CB, Woods L, Coyne-Beasley T, ACIP Adult Immunization Work Group, Centers for Disease Control and Prevention (CDC) (2013). Advisory committee on immunization practices (ACIP) recommended immunization schedule for adults aged 19 years and older - United States, 2013. MMWR Suppl.

[34] Ministério da Saúde (2023). Coqueluche.

[35] Gattás VL, Luna EJ, Sato AP, Fernandes EG, Vaz-de-Lima LR, Sato HK (2020). Ocorrência de eventos adversos após o uso da vacina adsorvida difteria, tétano e pertussis (acelular) - dTpa -, São Paulo, SP, 2015-2016. Epidemiol Serv Saúde.

[36] Chang MH (2007). Hepatitis B virus infection. Semin Fetal Neonatal Med.

[37] Boonyaratanakornkit J, Chu HY (2019). Why should we advocate maternal immunization?. Pediatr Infect Dis J.

[38] Li YT, Linster M, Mendenhall IH, Su YC, Smith GJ (2019). Avian influenza viruses in humans: lessons from past outbreaks. Br Med Bull.

[39] Hall E, Wodi AP, Hamborsky J, Schillie VM, Centers for Disease Control and Prevention (2021). Epidemiology and prevention of vaccine-preventable diseases.

[40] Moore MR, Link-Gelles R, Schaffner W, Lynfield R, Lexau C, Bennett NM (2015). Effect of use of 13-valent pneumococcal conjugate vaccine in children on invasive pneumococcal disease in children and adults in the USA: analysis of multisite, population-based surveillance. Lancet Infect Dis.

[41] Jarovsky D, Berezin EN (2023). Impact of PCV10 on pediatric pneumococcal disease burden in Brazil: time for new recommendations?. J Pediatr (Rio J).

[42] Strezova A, Diez-Domingo J, Al Shawafi K, Tinoco JC, Shi M, Pirrotta P (2022). Long-term protection against herpes zoster by the adjuvanted recombinant zoster vaccine: interim efficacy, immunogenicity, and safety results up to 10 years after initial vaccination. Open Forum Infect Dis.

[43] Kawai K, Gebremeskel BG, Acosta CJ (2016). Systematic review of incidence and complications of herpes zoster: towards a global perspective. BMJ Open.

[44] Kingsley T, Phelan D, Poland GA (2023). A review of 2023 adult immunization schedule updates. Vaccine.

[45] Godeaux O, Kovac M, Shu D, Grupping K, Campora L, Douha M (2017). Immunogenicity and safety of an adjuvanted herpes zoster subunit candidate vaccine in adults = 50 years of age with a prior history of herpes zoster: a phase III, non-randomized, open-label clinical trial. Hum Vaccin Immunother.

[46] Grohskopf LA, Blanton LH, Ferdinands JM, Chung JR, Broder KR, Talbot HK (2023). Prevention and control of seasonal influenza with vaccines: recommendations of the Advisory Committee on Immunization Practices - United States, 2023-24 Influenza Season. Centers for Disease Control and Prevention.

[47] Dunkle LM, Izikson R, Patriarca P, Goldenthal KL, Muse D, Callahan J (2017). Efficacy of recombinant influenza vaccine in adults 50 years of age or older. N Engl J Med.

[48] Siqueira JB, Massad E, Lobao-Neto A, Kastner R, Oliver L, Gallagher E (2022). Epidemiology and costs of dengue in Brazil: a systematic literature review. Int J Infect Dis.

[49] Lessa CL, Hodel KV, Gonçalves MS, Machado BA (2023). Dengue as a disease threatening global health: a narrative review focusing on Latin America and Brazil. Trop Med Infect Dis.

[50] Gallagher P, Chan KR, Rivino L, Yacoub S (2020). The association of obesity and severe dengue: possible pathophysiological mechanisms. J Infect.

[51] Patel SS, Rauscher M, Kudela M, Pang H (2023). Clinical safety experience of TAK-003 for dengue fever: a new tetravalent live attenuated vaccine candidate. Clin Infect Dis.

[52] El Hindi T, Anugulruengkitt S, Lapphra K, Tangsathapornpong A, Tsoukas CG, Hellwig M (2023). Immunogenicity and safety following co-administration of a live-attenuated tetravalent dengue vaccine and a recombinant 9-valent human papillomavirus vaccine in healthy participants aged ≥9 to <15 years in a dengue endemic country. Presented at the 72nd Annual Meeting of American Society of Tropical Medicine and Hygiene.

[53] Tricou V, Yu D, Reynales H, Biswal S, Saez-Llorens X, Sirivichayakul C (2024). Long-term efficacy and safety of a tetravalent dengue vaccine (TAK-003): 4·5-year results from a phase 3, randomised, double-blind, placebo-controlled trial. Lancet Glob Health.

[54] Ministério da Saúde (2021). Situação epidemiológica.

[55] Ministério da Saúde, Secretaria de Vigilância em Saúde e Ambiente, Departamento de Imunização e Doenças Transmissíveis (2023). Manual dos Centros de Referência para Imunobiológicos Especiais.

[56] Bruxvoort KJ, Lewnard JA, Chen LH, Tseng HF, Chang J, Veltman J (2023). Prevention of Neisserria gonorroeae with meningococcal B vaccine: a matched cohort study in Southern California. Clin Infect Dis.

[57] Marzochi KB, Homma A, Amato V (2011). Atualizações, orientações e sugestões sobre imunizações.

[58] Vizzotti C, Harris JB, Aquino A, Rancaño C, Biscayart C, Bonaventura R (2023). Immune response to co-administration of measles, mumps, and rubella (MMR), and yellow fever vaccines: a randomized non-inferiority trial among one-year-old children in Argentina. BMC Infect Dis.

